# A Text-Book of Physical Diagnosis

**Published:** 1903-10

**Authors:** 


A TEXT-BOOK OF PHYSICAL DIAGNOSIS. For students and Practitioners of Medicine. By Egbert Le Fevre, M. D., Professor of Clinical Medicine and Associate Professor of Therapeutics, University and Bellevue Medical College, Attending Physician to Bellevue and St. Luke's Hospitals, etc., New York. 12mo, 440 pages, with 74 engravings and 12 plates. Cloth, $2.25 net. Lea Brothers & Co., Philadelphia and New York.

Dr. Le Fevre is to be complimented for having so cleverly condensed so much valuable knowledge between the covers of his little text book on Physical Diagnosis. It is of special value to the student as well as the busy practitioner and is admirably fitted for review work. His directions in the methods of physical examination are full and explicit; the significance of same are dealt with in no small degree. Chapters directed to disease of the viscera contained in the thoracic cavity are especially worthy of note. The author also covers the latest results in X-ray diagnosis, including 12 full page plates. R. & L.

